# Interactions between body size, abundance, seasonality, and phenology in forest beetles

**DOI:** 10.1002/ece3.2732

**Published:** 2017-01-23

**Authors:** Mark A. K. Gillespie, Tone Birkemoe, Anne Sverdrup‐Thygeson

**Affiliations:** ^1^ Faculty of Environmental Sciences and Natural Resource ManagementNorwegian University of Life SciencesÅsNorway; ^2^ Department of Science & EngineeringWestern Norway University of Applied SciencesSogndalNorway

**Keywords:** beetles, dispersal, extinction risk, invertebrates, phenology, synchrony, traits

## Abstract

Body size correlates with a large number of species traits, and these relationships have frequently been used to explain patterns in populations, communities, and ecosystems. However, diverging patterns occur, and there is a need for more data on different taxa at different scales. Using a large dataset of 155,418 individual beetles from 588 species collected over 13 years of sampling in Norway, we have explored whether body size predicts abundance, seasonality, and phenology in insects. Seasonality is estimated here by flight activity period length and phenology by peak activity. We develop several methods to estimate these traits from low‐resolution sampling data. The relationship between abundance and body size was significant and as expected; the smaller species were more abundant. However, smaller species tended to fly for longer periods of the summer and peaked in midsummer, while larger species were restricted to shorter temporal windows. Further analysis of repeated sampling from a single location suggested that smaller species had increased flight period lengths in warmer years, but larger species showed the opposite pattern. The results 1) indicate that smaller species are likely to be disproportionately valuable in ecological interactions, and 2) provide potential insights into the traits influencing the vulnerability of some larger species to disturbances and climate change.

## 1 Introduction

The study of animal traits has contributed a great deal of insight into areas of ecology such as the organization of communities (Brown, Gillooly, Allen, Savage, & West, 2004; Brown & Maurer, 1987), the determination of relative species abundances and diversity (Siemann, Tilman, & Haarstad, 1996, 1999), and species distributions (Gaston & Lawton, 1988). For example, trait‐based approaches have been suggested as a useful way to predict extinction risk and the future impacts of habitat destruction (Fountain‐Jones, Baker, & Jordan, 2015; Kotiaho, Kaitala, Komonen, & Paivinen, 2005; Pedley & Dolman, 2014). Identifying the characteristics common to a wide range of key species is an important challenge in this approach, and the most useful trait relationships need to be tested for more taxonomic groups to further our understanding of their predictive capacity (Kotiaho et al., 2005). In this paper, we adopt a multi‐trait approach, utilizing a large database of beetle sampling in southern Norway to examine whether abundance, phenology, and seasonality can be effectively predicted by beetle body size. As body size is an easily obtainable species trait, demonstrating links with other life‐history traits can assist with conservation planning and designing future research.

The need for further research in this field is demonstrated by the lack of congruence in trait relationships across species groups. For example, while body size is fundamentally linked to metabolism and correlates with a large number of factors relevant to a species’ conservation status such as life span, habitat range, and abundance (Brown et al., 2004; Peters, 1983; Seibold et al., 2015), it is not important to the decline of all threatened species (Kotiaho et al., 2005). In addition, although the relationship between abundance and body size is among those most recognized and investigated (Davies, Margules, & Lawrence, 2000; Gaston, Blackburn, & Lawton, 1993; Green & Middleton, 2013; Siemann et al., 1999), generalizations and predictions are difficult because a wide range of correlations are documented (White, Ernest, Kerkhoff, & Enquist, 2007). The link between size and abundance is therefore a basic relationship that needs to be tested across all taxonomic groups.

While size and abundance are related to many traits, little is known about how they are associated with many other aspects of species ecology. For example, phenology, the timing of life‐history events such as adult emergence, is one of the aspects of species ecology that helps to determine species coexistence, species interactions, and community structure (Nieminen, 2015; Pozsgai & Littlewood, 2014). For many species, phenology has been shown to change over time in response to warming global temperatures (Root et al., 2003), and for mammals, body size has been found to scale positively with phenological sensitivity to climate change (McCain & King, 2014). However, a corresponding link for insects has not been investigated. Insect body size may be important in this respect because it can define the microclimatic niche occupied by the species and therefore determines how a species interacts with its environment (McCain & King, 2014).

Closely related to a species’ phenology is its seasonality: the degree of phenological synchronization within populations. This is likely to be another trait susceptible to global changes. For example, Ribeiro and Freitas (2011) found that larger butterfly species were highly seasonal and restricted to narrower “temporal windows” of adult activity than smaller species. They suggest that this places large species at risk from disturbance due to asynchrony with key resources. Furthermore, if the sub‐populations of a species are synchronous across a landscape, this can promote instability at the meta‐population level in certain situations (Abbott, 2011). Small sub‐populations migrating at the same time will remain simultaneously small across their range, putting the species at risk of extreme perturbations. As population synchrony can also increase with higher temperatures in some species (Illan, Gutierrez, Diez, & Wilson, 2012; Zografou et al., 2015), there may be an important link between climate, phenology, and seasonality and a species’ body size and relative abundance.

In this study, we estimate the length (seasonality) and peak (phenology) of the flight activity period for species in a large database consisting of 588 beetle species and 155,418 individuals collected in traps mounted on elements of dead wood in southern Norway between 2001 and 2013. We aimed to use this dataset to test whether our phenology and seasonality variables interact with body size and abundance. Due to the current theory and empirical evidence outlined above, we hypothesized that body size would scale negatively with abundance in line with findings for many other species groups. Furthermore, in line with the findings on butterflies, we expect larger species to have longer flight activity periods. The importance of summer length for flight activity periods and peaks is also tested here for a subset of species.

## 2 Materials and Methods

### 2.1 Study sites and sampling

**Figure 1:**
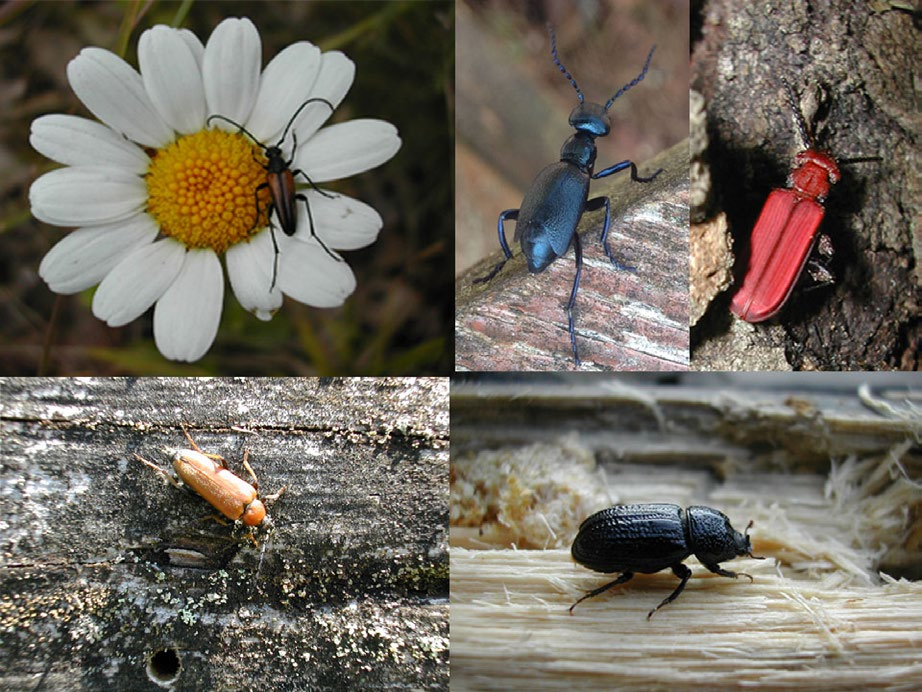
Examples of the study organisms, saproxylic beetles that depend on dead or decaying wood in forests. Photographs: Anne Sverdrup‐Thygeson

Beetles were collected from 37 sites in southern Norway (Fig. S1) over a period of 13 years (2001–2013). A full list of sites and their coordinates and the trapping effort associated with these sites are found in Table S1. The details of the study sites and trapping methodology have been given elsewhere (Birkemoe & Sverdrup‐Thygeson, 2015; Fossestøl & Sverdrup‐Thygeson, 2009; Gough, Birkemoe, & Sverdrup‐Thygeson, 2014; Sverdrup‐Thygeson, 2002; Sverdrup‐Thygeson, Bendiksen, Birkemoe, & Larsson, 2014; Sverdrup‐Thygeson & Ims, 2002; Sverdrup‐Thygeson, Skarpaas, & Odegaard, 2010), but are briefly summarized here. Sampling was conducted using flight interception traps (with crosspane windows sized either 20 × 40 cm or 40 × 60 cm), a funnel, and a container underneath filled with either ethylene glycol or propylene glycol and detergent. The traps were mounted in forest sites spanning the typical forest types in southern and southeastern Norway, with dominant species of Norwegian spruce (*Picea abies*), Scots pine (*Pinus sylvestris*), birch (*Betula pubescens* or *Betula pendula*), aspen (*Populus tremula*), and oak (*Quercus petrea* or *Quercus robur*). The traps were mounted in May and emptied monthly until late August. All beetle individuals were identified to species level, and scientific names are in accordance with the Norwegian Species Nomenclature Database. The classification of saproxylic species (Figure 1) was based on relevant literature, mainly (Dahlberg & Stokland, 2004). Prior to analysis, species with less than 10 individuals and/or caught in less than five traps were removed from the dataset. Analyses were subsequently conducted on all remaining 588 species and on a subset consisting of the 420 saproxylic species in the dataset. To test for the effect of annual variation in temperature, we selected one site from the database with records from the most number of years (7). The above criteria were applied to the data from this site, and this resulted in a subset of 77 species. Only one site was used for this analysis because the captured species, sampling dates, and sampling effort differed widely between sites. Furthermore, sites varied in their distance to reliable weather stations, so a combined analysis of all sites would have been based on weather data of varying accuracy.

### 2.2 Body size

Mean body sizes in millimeters were collated for all species in the dataset from a range of sources. Firstly, where available, sizes were taken from Gossner et al. (2013) and Seibold et al. (2015). However, for the remaining 302 species, body size ranges were taken from identification keys available online (Die Käfer Europas: www.coleo-net.de [190 species]; www.zeno.org [70 species]) or in published works (see Table S2.1 in Supporting Information for a full list). Sizes were most often expressed as a range, so the mean of the maximum and minimum sizes was calculated and used as “body size.”

### 2.3 Abundance

The dataset used in this study is characterized by an unbalanced sampling history: Some sites were sampled more often than others, and as a result, the raw abundance data may be biased. Presence or count data may also suffer from this problem; for example, southern restricted species may appear more abundant or “present” if there were more sampling events in the south. To account for this bias, we standardized the abundance data by calculating the proportion of sampling occasions in which a species was caught at each site. For example, if a species was present in five trapping occasions out of a total of 30 possible trapping occasions, the “proportional abundance” of this species at this site is 0.167 (5 ÷ 30). The overall proportional abundance of a species was the sum of all site proportions.

### 2.4 Phenology and seasonality estimations

Precise estimates for phenology and seasonality could not be calculated from this dataset because of the large trapping intervals (overall mean = 31.6 ± 6.1 s.d. days) and variable trapping dates between sampling years. Despite this, three trapping “phases” could clearly be distinguished from the full dataset with only a few days of overlap between years. These phases can be described as “early summer” (trapping begins between 2 and 22 May and ends between 4 and 25 June, mean trapping period = 33.1 ± 5.0 s.d. days), “midsummer” (trapping begins 5–26 June to 7–30 July, mean = 28.8 ± 3.9 s.d. days), and “late summer” (trapping begins 7–31 July and ends in 6–28 August, mean = 30.6 ± 3.4 s.d.days). We calculated flight activity period (seasonality) using species presence data during these phases. We used species presence instead of total abundance because the type of traps used can often capture a disproportionate number of a single species in a single trapping session, leading to results bias (Gossner et al., 2013).

Our flight activity period variable is based on proportions trapped in the different phases and is an adaptation of the classifying scheme for aquatic beetles devised by Boda and Csabai (2013). The proportion *p* of all trapping occasions (species present in a trap) of species *i* occurring in each time phase *j* (early, mid, or late summer) was calculated, and the phase with the highest proportion was regarded as the “global peak” (*p*_max_, sensu Boda & Csabai, 2013). The proportions of the remaining two phases were then expressed as a percentage of the global peak (*p*_*j*_/*p*_max_). Thus, the smaller the proportion of individuals dispersing in the early summer phase for example, the more likely that the activity period begins close to the start of the midsummer phase. In this instance therefore, only a small number of days at the end of the early summer phase will contribute to the total activity period. To calculate the likely number of days of flight activity in each phase, the adjusted proportions were multiplied by the mean number of days of trapping for the corresponding phase *l*_*j*_. The sum of the number of days of flight activity for each of the three phases then represents the total flight activity period in days (Equation (1))

(1) $$\text{Flight Activity}\;{\text{Period}}_{i}=\sum \left(\frac{p_{j}}{p_{\max}}l_{j}\right),$$

where

(2) $$l_{j}=\frac{\sum (e_{k}-s_{k})}{n},$$

where *e* is the Julian day that trap *k* (a trap that caught one or more of species *i*) was collected and *s* is the Julian day that trap *k* was set up.

Peak flight dates (phenology) were estimated using the flight activity period data. First, we calculated the mean last day of trapping for the first phase in which the species was caught. From this date, we subtracted the estimated number of days for that first phase, calculated above. This represented the estimated first day of flight. Assuming that population densities followed a triangular function, half of the flight activity period was added to this date to estimate the peak activity date.

We also tested three alternative methods of calculating flight activity period and peak activity (Appendices S2 and S3). The resulting estimates were all well correlated with our chosen method and had similar results in mixed modeling analysis. We prefer the chosen method because the calculated variables are not directly derived from abundance or species presence. Commonness is one of the main explanatory terms of interest, so it is important that the response variables are as independent as possible from abundance.

### 2.5 Distribution

To take account of geographical variability, weighted means and weighted standard deviations of latitude and longitude were calculated for each species. As not all species were captured in all traps and at all sites, the means were derived to act as a proxy for the center of the range of each species. Similarly, the standard deviations were regarded as proxies for the extent of the distributional range of each species. As a simple mean may be skewed due to varying sampling effort between sites and years, a weighted mean and standard deviation were calculated as:

(3) $$\overset{¯}{x}=\frac{\sum (w/c)wx}{\sum (w/c)w},$$

(4) $$SD=\frac{\surd \sum \left(\left(w/c\right)w{\left(x-\overset{¯}{x}\right)}^{2}\right)}{\sum (w/c)w},$$

where *w* is the number of times a species was caught at latitude (or longitude) *x*, and *c* is the number of sampling sessions at that latitude (or longitude).

### 2.6 Data analysis

We used linear mixed effects modeling using the *mgcv* package (Wood, 2011) of the R programming environment (R Core Team 2014). The first models used proportional abundance as response variable, and body size and geographical variables as explanatory variables. The second and third set of models were analyzed with flight activity period (seasonality) and peak flight date (phenology), respectively, as response and body size, proportional abundance, and geographical variables as explanatory terms. The interaction between body size and abundance was included, but removed if it was nonsignificant. The explanatory variables as body size and proportional abundance were logarithmically transformed to reduce the influence of extreme values. Of the geographical variables, latitude and latitudinal range were better explanatory covariates than the longitude variables, as measured by AIC. Therefore, only the effects of the “Latitude models” are presented. As the use of closely related species as observations violates the assumption of independence, the categorical variable of Family was used first as a fixed term. However, Family did not have a significant effect in any of the models and the impact of the factor on model fit (as measured by AIC) was detrimental largely because there are representatives of 58 families in the dataset, some with only 1–5 members. The families with less than 10 members were therefore grouped by superfamily, resulting in a factor with 26 levels. Again this family/superfamily amalgamation factor (hereafter “Family2”) had no significant effect and the AIC values were only slightly improved, so Family2 was included as a random factor.

The final models were based on a subset of the data from a study area where we had repeated sampling of over 40 sites for 7 years (2001–2005, 2007, and 2013), combined with temperature data to evaluate how variation in annual temperature accumulation influences the variation in seasonality and phenology. These models had flight activity period and peak flight date as response variables and body size, count of species presence, and cumulative growing degree days (GDDs) as explanatory variables. GDD is a measurement of heat accumulation with a history of use in agriculture to predict crop and pest phenology (Parry & Carter, 1985), and more recent use in ecological studies of climate and phenology (Cayton, Haddad, Gross, Diamond, & Ries, 2015; Hodgson et al., 2011). For example, annual GDD can be used as a measure of temporal variation in warmth available to organisms: In warm years, heat accumulates faster and the total number of GDD is higher (Hodgson et al., 2011). In this study, GDD was calculated using daily maximum and minimum temperature data taken from the Ås weather station approximately 23 km from the sampling site (Norwegian Meteorological Institute, www.eklima.no) from 1 January to 31 December each year. We also calculated GDD for the main flight period (until 31 August, the last trap collection date) in case cool autumn temperatures affected the annual GDD. The base temperature for both GDDs was set at 10°C and the maximum temperature at 30°C. These are commonly used thresholds for phenological prediction of insect pests, including beetles (Nufio, McGuire, Bowers, & Guralnick, 2010), and were used here in the absence of known thermal tolerance limits for forest beetles (sensu Cayton et al., 2015). The GDD × body size interaction was also included in the models to evaluate the extent to which size impacts the response of flight activity period to temperature accumulation. Year (factor), Family, and species were included as random factors as these best accounted for the autocorrelation in the data according to a comparison of AIC values and autocorrelation function plots of the residuals. All models were subjected to postanalysis checks for heteroscedasticity, influential observations, and non‐normality of residuals.

## 3 Results

### 3.1 Abundance and body size

**Figure 2:**
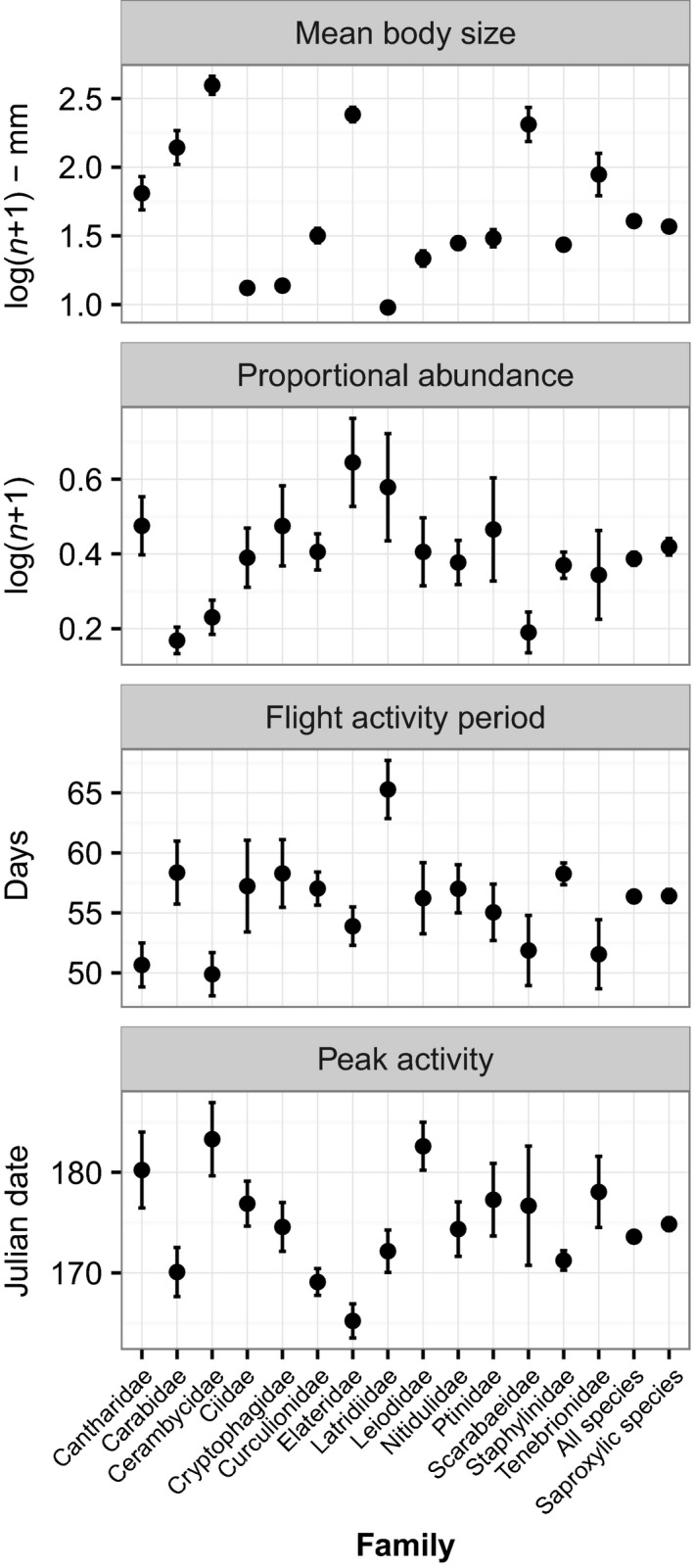
Four variables calculated for each of the species, presented as means for the main families (those with 10 or more members in the dataset), together with the means of all species taken together and of saproxylic species only. The error bars are 1 *SE*

**Figure 3:**
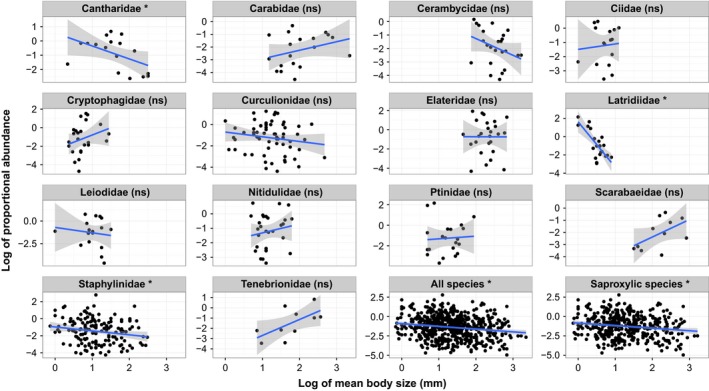
Proportional abundance (log transformed) plotted against body size (log transformed) for families with more than 10 species in the database (in alphabetic order), as well as all species in the dataset and all saproxylic species. The blue line represents the linear model of the relationship, and the gray area depicts 95% confidence intervals. Note that the *y*‐axis scale differs between panels. The only significant within family correlations are for the Cantharidae, Latridiidae, and Staphylinidae. (ns = nonsignificant)

There was a range of body sizes and abundances represented in the dataset (Figure 2). Within the main families, body size in particular varied only slightly among the species. In terms of correlations between these two variables, there was a range of trends for the largest families (Figure 3), but only the correlations for three families (Cantharidae [Pearson's product–moment correlation: *t* = −2.15, *df* = 15, *p* = .048], Latridiidae [*t* = −4.89, *df* = 19, *p* = .0001], and Staphylinidae [*t* = −2.28, *df* = 150, *p* = .024]) were significant. When analyzed together with geographical variables, proportional abundance was strongly and negatively correlated with body size for the dataset including all species and for the subset of saproxylic species (Table 1).

**Table 1:** Parameter estimates of the two linear mixed models performed using the full dataset and the subset of Saproxylic species, for the “proportional abundance” response variable

	All species (*n* = 588)			Saproxylic species (*n* = 420)		
	Effect ± *SD*	*t*	*p*	Effect ± *SD*	*t*	*p*
Fixed effects						
Intercept	48.4 ± 5.4	8.96	<.001	44.9 ± 6.2	7.54	<.001
Size (log transformed)	−0.3 ± 0.1	−3.68	<.001	−0.3 ± 0.1	−2.83	.005
Latitude	−0.8 ± 0.1	−9.19	<.001	−0.8 ± 0.1	−7.75	<.001
Latitudinal range	1.3 ± 0.2	5.20	<.001	1.3 ± 0.3	4.61	<.001
*df*	557			393		
Random effects						
Intercept	0.19			0		
Family^a^	0.005			<0.001		
*R* ^2^‐adj	.16			.16		

a The factor “Family,” an amalgamation of family and superfamily, was used as random factor, and effects given are standard deviations across groups.

### 3.2 Seasonality and body size

**Figure 4:**
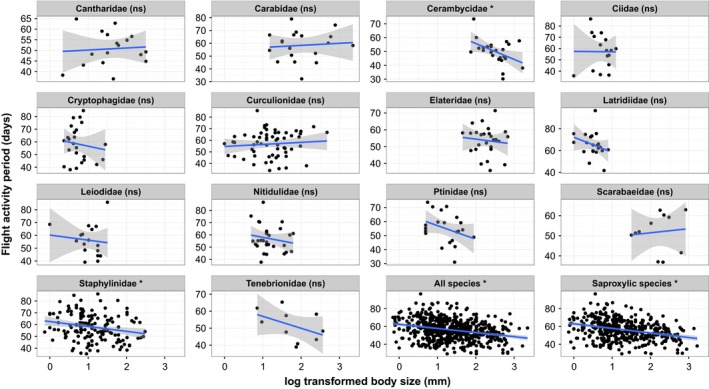
Flight activity period (seasonality) plotted against body size (log transformed) for families with more than 10 species in the database (in alphabetic order), as well as all species in the dataset and all saproxylic species. The blue represents the linear model of the relationship, and the gray area depicts 95% confidence intervals. Note that the *y*‐axis scale differs between panels. The only significant within family correlations are for the Cerambycidae and Staphylinidae. (ns = nonsignificant)

Seasonality as defined by flight activity period ranged from 29.33 to 96.6 days with a mean of 56.37 ± 11.45 s.d. days, and a range of values were found between and within the major families (Figure 2). There were negative relationships between seasonality and body size for the main families except the Cantharidae and Curculionidae (Figure 4), although the only significant correlations were for the Cerambycidae (*t* = −2.41, *df* = 22, *p* = .025) and the Staphylinidae (*t* = −2.70, *df* = 150, *p* = .007). Models with flight activity period as response variable produced similar results for the dataset including all species and for the dataset including saproxylic species only (summarized in Table 2). There was a consistent strong positive effect of proportional abundance and a highly significant negative effect of body size on the flight activity period of all species and saproxylic species. There was no interaction between size and abundance.

**Table 2:** Parameter estimates of the two linear mixed models performed using the full dataset and the subset of Saproxylic species, for the flight period length response variable

	All species (*n* = 588)			Saproxylic species (*n* = 420)		
	Effect ± *SD*	*t*	*p*	Effect ± *SD*	*t*	*p*
Fixed effects						
Intercept	28.1 ± 45.0	0.62	.533	14.4 ± 50.9	0.28	.778
Proportional abundance (log transformed)	2.1 ± 0.3	6.57	<.001	2.1 ± 0.4	5.45	<.001
Size (log transformed)	−3.7 ± 0.6	−5.76	<.001	−4.4 ± 0.7	−6.04	<.001
Latitude	−0.6 ± 0.8	0.76	.451	0.8 ± 0.9	0.95	.345
Latitudinal range	3.8 ± 2.0	1.95	.052	3.8 ± 2.3	1.68	.094
*df*	556			392		
Random effects						
Intercept	0.40			0		
Family^a^	0.001			<0.001		
*R* ^2^‐adj	.15			.17		

a The factor “Family,” an amalgamation of family and superfamily, was used as random factor, and effects given are standard deviations across groups.

### 3.3 Phenology and body size

Phenology as defined by peak activity date ranged from Julian day 145.5 (c. 24 May) to 225.6 (c. 12 August) with a mean of 173.6 ± 13.4 s.d., and a range of values were found between and within the major families (Figure 2). Models with peak date as response variable produced very low *R* ^2^ values (summarized in Table 3), so should be viewed with caution. For the “all species” dataset, there was a weak positive effect of proportional abundance on peak activity date. The weakly significant interaction term suggests that large and less abundant species peak late in the season, while large and more abundant species peak early in the season. The opposite appears to be the case for small species, but the variability in peak activity is reduced for these species. For the saproxylic dataset, the same effects were found, but with a stronger interaction effect. In addition, there was a weak negative relationship between peak date and body size, indicating that larger species tend to peak earlier in the season.

**Table 3:** Parameter estimates of the two linear mixed models performed using the full dataset and the subset of Saproxylic species, for the peak activity date response variable

	All species (*n* = 588)			Saproxylic species (*n* = 420)		
	Effect ± *SD*	*t*	*p*	Effect ± *SD*	*t*	*p*
Fixed effects						
Intercept	403.2 ± 55.6	7.25	<.001	358.3 ± 63.3	5.66	<.001
Proportional abundance (log transformed)	1.7 ± 0.8	2.08	.038	2.0 ± 0.9	2.26	.024
Size (log transformed)	−2.1 ± 1.2	−1.69	.092	−3.3 ± 1.3	−2.50	.013
Latitude	−3.8 ± 0.9	−4.07	<.001	−3.0 ± 1.1	−2.82	.005
Latitudinal range	4.2 ± 2.4	1.76	.079	2.9 ± 2.7	1.05	.293
Abundance × size	−1.3 ± 0.5	−2.46	.014	−1.7 ± 0.6	−2.91	.004
*df*	555			391		
Random effects						
Intercept	4.50			3.48		
Family^a^	0.12			0.08		
*R* ^2^‐adj	.05			.04		

a The factor “Family,” an amalgamation of family and superfamily, was used as random factor, and effects given are standard deviations across groups.

### 3.4 Annual variability with temperature

**Figure 5:**
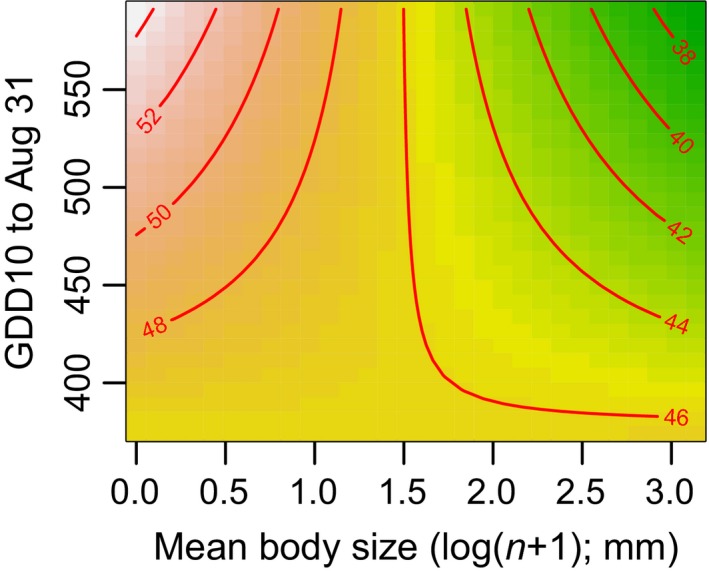
Contour map of model predictions for the Østmarka dataset to demonstrate the interaction between growing degree days (GDD10) and body size. The contour lines and shading depict the flight activity period in days (response variable). Therefore, the model predicts that during warmer summers, the flight activity period of small species will increase, but for larger species it will decrease

The best time‐series model in terms of explanatory power (adj‐*R* ^2^) used the GDD with a baseline of 10°C and a timeframe of 1 January to 31 August. This model revealed a weak significant interaction between GDD and body size (*p* = .024; Table S4), indicating that species of different sizes respond differently to temperature in terms of activity period. This interaction is best depicted in Figure 5, showing that smaller species have longer flight period lengths (less seasonality) in warmer years, whereas larger species have a shorter flight period in warmer years. It should be noted, however, that this result depends on the GDD used, with no significant interaction for GDD for the whole year, or when using a baseline temperature of 5°C. There were no significant effects of GDD and size on phenology.

## 4 Discussion

In this study, saproxylic and other beetles of small‐to‐intermediate body sizes were found to be more abundant than large species. This result was expected as it is a well‐established pattern for many animal communities elsewhere (Gaston, Blackburn, Hammond, & Stork, 1993; Gaston, Blackburn, & Lawton, 1993). For forest beetles, the relationship can be linked to niche requirements. Larger saproxylic beetle species tend to prefer larger trees and dead wood at late stages of decay (Brin, Bouget, Brustel, & Jactel, 2011; Gossner et al., 2013) that provide a more stable environment for the long larval development time (Foit, 2010). Conversely, smaller species should theoretically be able make use of a wider range of niche sizes (Blackburn & Gaston, 1997), including both small and large woody objects. Furthermore, mean body length of beetles tends to increase with the diameter of deadwood (Brin et al., 2011), resulting in larger dead wood objects accommodating more species (Grove, 2002; Jonsell, Weslien, & Ehnstrom, 1998). As the number of large hollow trees and the amount of large‐sized dead wood is declining in Europe, habitats providing suitable niches for larger wood‐living species are also likely to be on the decline. It is perhaps unsurprising, therefore, that large saproxylic beetle species are rarer and more susceptible to extinction (Brin, Valladares, Ladet, & Bouget, 2016; Davies et al., 2000; Seibold et al., 2015).

We also found relationships between body size and other life‐history traits. Firstly, we found that large and less abundant species tended to occur late in the summer, but more abundant large species were active earlier. This pattern may occur because of dispersal strategies: Species dispersing early in the season may exhibit faster population growth because they have a longer period in which to successfully mate or lay eggs, or to consume sufficient resources required to survive the winter (Pozsgai & Littlewood, 2014). Previous studies have demonstrated similar patterns for other species groups and also show that some late‐flying species tend to emerge later in response to increasing temperatures, compared to early emerging species that advance their emergence phenology (Altermatt, 2010; Pozsgai & Littlewood, 2014; although see Zografou et al., 2015). Our data are not sufficient to investigate this latter tendency for our study species, and the explanatory power of our phenological models was weak. However, the finding that body size, abundance, and phenology are linked does indicate that future research into the effects of changing temperatures on forest beetle phenology is warranted.

Secondly, we found that the flight activity period of smaller species tends to be longer than that of the larger species. This pattern is likely to be a reflection of the adult life spans of beetle species. For example, the Cerambycidae represent the taxonomic group with the largest species in the current dataset and are known to have short adult life spans, whereas small bark beetles have comparatively longer adult life lengths (Ehnstrom & Axelsson, 2002). Alternatively, larger species may be more synchronous in their flight and dispersal. This is the case in other species groups, for example in butterflies (Ribeiro & Freitas, 2011; although see Franzen & Betzholtz, 2012), suggesting a trade‐off between insect size and seasonality. One reason for these patterns could be that larger forest beetle species require optimal environmental conditions to initiate dispersal and that the high‐energy demands of flight for these species constrain the length of the flight period. The limited range of optimal environmental conditions for large species could make them “temporal specialists,” susceptible to changes in weather and habitat fragmentation (Ribeiro & Freitas, 2011). For example, models of size–density relationships and resource distribution predict that large‐bodied species will be more sensitive when environmental change results in resources becoming fragmented and sparse (Nilsen, Finstad, Naesje, & Sverdrup‐Thygeson, 2013). A short temporal window of adult flight activity is likely to make it more difficult for large species to find fragmented patches of resources.

Our strongest negative relationship between body size and seasonality was found when analyzing saproxylic species only. This relationship may relate to a saproxylic lifestyle, therefore. Dead wood undergo large successional changes, from the first and very short nutrient‐rich stage when the cambium is still present to the final long‐lasting stages of decomposing heartwood. Beetles exploiting the first successional stage tend to have a shorter larval development time and smaller body size than species in later succession (Kletecka, 1996). This is supported by the fact that nutritional variation or diet combined with microhabitat use has been suggested as the primary driver for body size in canopy invertebrates and beetles, respectively (Barton, Gibb, Manning, Lindenmayer, & Cunningham, 2011; Wardhaugh, Edwards, & Stork, 2013). As early successional stages in wood decay also are more ephemeral than later stages, differences in dispersal frequency are expected (Nilsson & Baranowski, 1997; Southwood, 1977). Small, early‐decay species with good dispersal abilities are therefore likely to be followed by larger species with lower dispersal abilities during the succession of wood decomposition.

There may be advantages and disadvantages of greater seasonality for large species. For example, less abundant species emerging in relative synchrony are likely to encounter mates more often than if their flight is spread across the season. However, the more abundant and less seasonal small‐ and intermediate‐sized species are more likely to exhibit stability within meta‐populations in the event of variable weather events and other perturbations, because neighboring populations may be dispersing during different overlapping time periods (Abbott, 2011; Nieminen, 2015). Furthermore, in this study smaller species tended to increase the length of the flight activity period during warmer years, perhaps due to an increase in bivoltinism (Altermatt, 2010). Conversely, larger species reduced the flight period during warm years, implying that they will have a tendency to become more synchronized in a warmer world, further constraining their ability to stabilize meta‐populations and track fragmented resources. However, more studies are required to investigate this further, and as with the phenology analyses, the interaction between size and GDDs was weak.

In summary, we found that small‐ and intermediate‐sized forest beetles tended to be more abundant and have longer summer flight activity periods, and this may suggest that they play disproportionally large roles in ecological interactions in forests. Conversely, larger species tended toward lower abundance and greater seasonality or “temporal specialization.” Large species also tend to exhibit lower population growth rates (Lawton, Daily, & Newton, 1994) and habitat specialization making them susceptible both to habitat destruction and climate change (Rainio & Niemela, 2003; Ribeiro & Freitas, 2011). Thus, our findings may demonstrate some added challenges for some large species and highlight further traits that may influence their vulnerability. The parameters used here, body size and flight activity period, and to a lesser extent peak activity date, have the advantage of being easily attainable, which should stimulate more research into this field. Further research is required to test the use of these traits for more taxonomic groups, to elucidate the causal mechanisms of the negative size–seasonality relationship, and to test the level of plasticity of flight activity period for small vs large species.

## Conflict of Interest

None declared.
