# Supplementary material for: Interactions between body size, abundance, seasonality, and phenology in forest beetles
Source: Ecol Evol. 2017 Jan 23;7(4):1091–100. doi: 10.1002/ece3.2732 (PMC5306008; doi:10.1002/ece3.2732)
Supplement: Supplementary file 2 [file ECE3-7-1091-s002.docx]

**Supporting Information**

**Appendix S1: Map and co-ordinates of field sites**


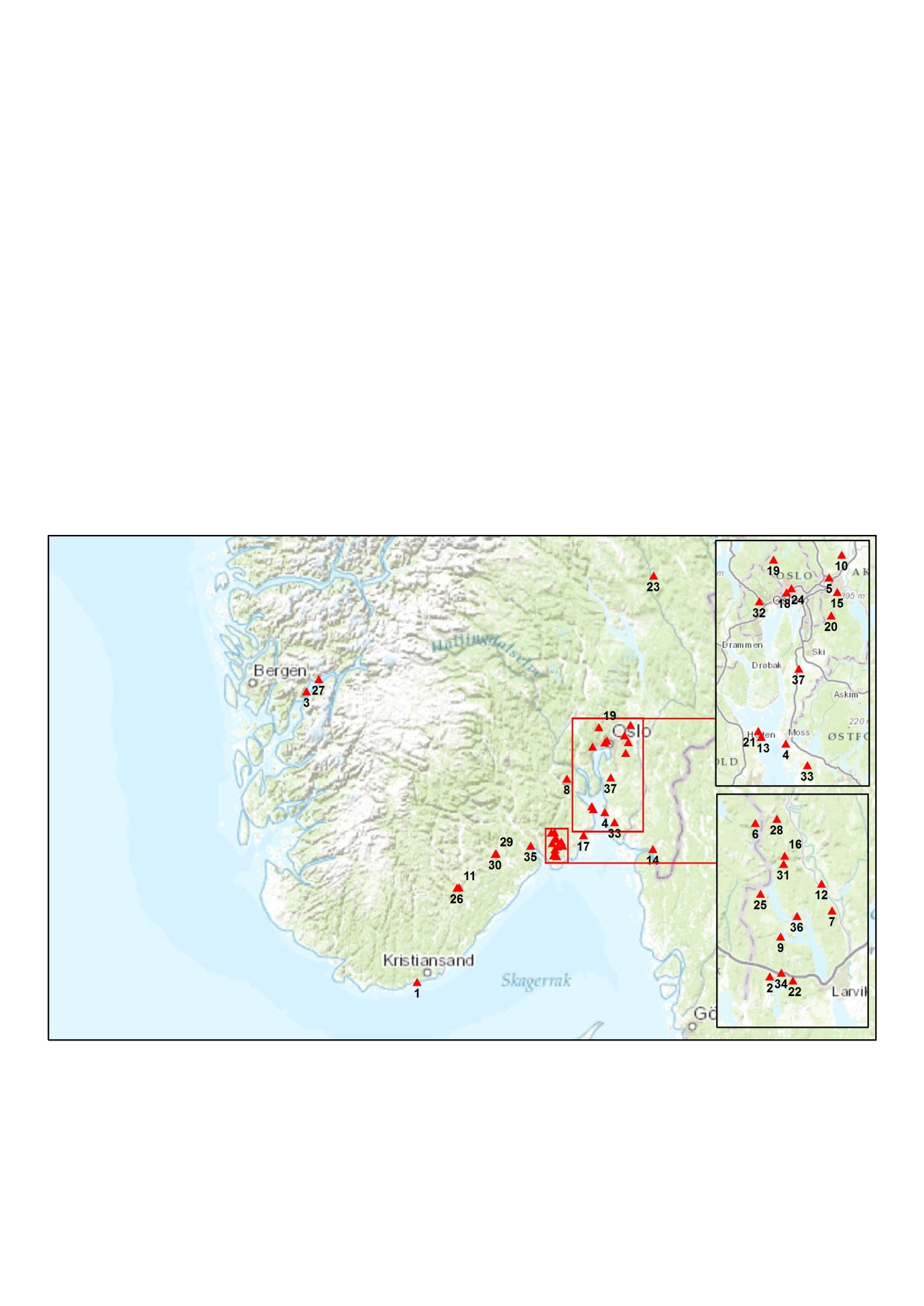


Figure S1: Map showing the field sites from the study. The numbered labels correspond to the site numbers in table S1 below.

Table S1: Field sampling sites used in the study, their coordinates and sampling time.

| **Site Number** | **Site name** | **Longitude** | **Latitude** | **Number of years sampled** | **Total days sampled** | **Mean sampling days per year** |
| --- | --- | --- | --- | --- | --- | --- |
| 1 | Årosveten | 7.833 | 58.069 | 4 | 353 | 88 |
| 2 | Askedalsåsane | 9.891 | 59.065 | 1 | 103 | 103 |
| 3 | Berge LV | 6.166 | 60.312 | 1 | 95 | 95 |
| 4 | Bog (ved Carlberg) | 10.663 | 59.398 | 1 | 100 | 100 |
| 5 | Bøler (Gjelleråsen) | 10.965 | 59.983 | 1 | 100 | 100 |
| 6 | Brenndalsskarven | 9.859 | 59.240 | 5 | 478 | 96 |
| 7 | Budalsåsen | 10.029 | 59.140 | 5 | 476 | 95 |
| 8 | Drammensmarka | 10.093 | 59.655 | 1 | 114 | 114 |
| 9 | Eikvang | 9.915 | 59.110 | 1 | 104 | 104 |
| 10 | Fjellstad | 11.051 | 60.062 | 1 | 100 | 100 |
| 11 | Gangseid | 8.470 | 58.815 | 1 | 81 | 81 |
| 12 | Gjønnesvannet | 10.006 | 59.171 | 1 | 102 | 102 |
| 13 | Karljohansvern | 10.491 | 59.423 | 4 | 381 | 95 |
| 14 | Knardal | 11.390 | 59.115 | 4 | 269 | 67 |
| 15 | Kurland | 11.020 | 59.932 | 1 | 100 | 100 |
| 16 | Kvelderønningen | 9.924 | 59.202 | 1 | 103 | 103 |
| 17 | Melsomvik | 10.344 | 59.220 | 4 | 380 | 95 |
| 18 | Montebello | 10.668 | 59.931 | 4 | 327 | 82 |
| 19 | Nordmarka | 10.576 | 60.045 | 6 | 548 | 91 |
| 20 | Østmarka | 10.979 | 59.851 | 7 | 633 | 90 |
| 21 | Østøya | 10.471 | 59.444 | 1 | 98 | 98 |
| 22 | Pauler | 9.942 | 59.061 | 1 | 105 | 105 |
| 23 | Rena leir | 11.403 | 61.167 | 1 | 92 | 92 |
| 24 | Ris (Risbakken 22) | 10.700 | 59.944 | 1 | 100 | 100 |
| 25 | Sandvikskollane | 9.870 | 59.159 | 1 | 105 | 105 |
| 26 | Simonstona | 8.433 | 58.812 | 4 | 367 | 92 |
| 27 | Skeianes | 6.347 | 60.404 | 1 | 96 | 96 |
| 28 | Søndre Odbergsetra (Rimstad) | 9.906 | 59.244 | 1 | 106 | 106 |
| 29 | Steinknapp Øst | 9.024 | 59.081 | 4 | 362 | 91 |
| 30 | Steinknapp Vest | 9.012 | 59.077 | 1 | 99 | 99 |
| 31 | Store Limtjønn | 9.922 | 59.193 | 1 | 104 | 104 |
| 32 | Tanum | 10.478 | 59.899 | 1 | 99 | 99 |
| 33 | Tomb | 10.812 | 59.321 | 1 | 100 | 100 |
| 34 | Vassbotten | 9.916 | 59.069 | 1 | 105 | 105 |
| 35 | Veholt | 9.549 | 59.140 | 1 | 104 | 104 |
| 36 | Vemannsås | 9.951 | 59.134 | 5 | 440 | 88 |
| 37 | Vollebekk | 10.754 | 59.663 | 1 | 100 | 100 |

**Appendix S2: Description of alternative flight activity length variables**

Below is a description of the calculation method for three variables for the flight activity period length which could be alternatives to the variable used in the main text. The data for the variables can be found in Table S2.1 and the correlation coefficients between the variables are shown in Table S2.2 below.

1. *Trapping length*

The variable “trapping length” is rather simple and does not directly take into account the presence/absence of a species. First, a mean of the first day of all trapping occasions in which a species was caught in each phase was calculated, expressed as Julian day. The earliest of these was treated as the start of the flight activity period (i.e., if a species was not caught in early summer, the mid-summer mean start day was treated as the start date). Secondly, the mean last day of all trapping occasions in which a species was caught in each phase was calculated, and the latest of these was regarded as the end day of the flight activity period. The Trapping Length is then the end day minus the start day.

1. *Weighted month standard deviation*

The second variable, “weighted month standard deviation” (hereafter WMSD), is an adaptation of a common method used for much finer resolution data (i.e., daily or weekly sampling) (e.g. Bishop et al. 2013). Here, the mean flight date describing the timing of flight is calculated by the weighted mean:

$\bar{x}= \sum wx/\sum w$ [3]

and the flight period length is represented by the corresponding standard deviation:

$SD=\surd\sum(w(x-\bar{x})^{2})(\sum w/\sum(w)^{2}-\sum w^{2})$ [4]

where $x$ is the number of days since some starting date and $w$ is the total number of individuals caught per day. In this adapted version, $x$ is the end month of the trapping phase expressed as a number (e.g. June = 6) and $w$ is the number of times a species was present in a trap in that phase. This standard deviation is therefore expressed in months and the resulting metric ranged from 0.24 to 1.07 months.

1. *Seasonality index*

This measure has been used for butterflies (Ribeiro & Freitas 2011) and uses the length of the mean vector (*r*) from circular statistics as the index (Zar, 1999):

$r= \frac{\sqrt{(\sum_{i=1}^{n} \sin a)^{2}+(\sum_{i=1}^{n} \cos a)^{2}}}{n}$ [3]

where, $a=\frac{starting month \times360^{\circ}}{12}$ [4]

For our data, the starting month could only take one of three values: 5, 6 or 7 which correspond to the months that trapping started in each trapping phase (May, June and July). The index can take a value between 0 and 1, where 0 indicates that the probability of a species occurrence is the same for each of the 3 trapping phases (i.e., a long flight activity period), and 1 indicates that all individuals of the species occur in the same phase (i.e., a short flight activity period). 3700.58.18673

**Table S2.2:** Correlation matrix of the three flight activity period variables for all species and saproxylic species only. Correlation coefficients are Pearson product moment coefficients (ρ). Flight activity period = response variable used in main text, WMSD = weighted month standard deviation, , SI = seasonality index.

|  | All species | | | Saproxylic species only | | |
| --- | --- | --- | --- | --- | --- | --- |
|  | SI | WMSD | Trapping Lengh | SI | WMSD | Trapping Length |
| Flight activity period | -0.75 | 0.71 | 0.42 | -0.72 | 0.68 | 0.37 |
| SI | - | -0.88 | -0.56 | - | -0.88 | -0.53 |
| WMSD | - | - | 0.70 | - | - | 0.68 |

**References**

Bishop, T.R., Botham, M.S., Fox, R., Leather, S.R., Chapman, D.S. & Oliver, T.H. (2013). The utility of distribution data in predicting phenology. *Methods in Ecology and Evolution, 4, 1024-1032.*

Ribeiro, D.B. & Freitas, A.V.L. (2011). Large-sized insects show stronger seasonality than small-sized ones: a case study of fruit-feeding butterflies. *Biological Journal of the Linnean Society*, 104, 820-827.

Zar, J.H. (1999) Biostatistical Analysis. Prentice Hall, New Jersey.

**Appendix S3: Model output from Trapping Length, WMSD and Seasonality index response variables.**

Table S3.1: Parameter estimates of the two linear mixed models performed using the full dataset and the subset of Saproxylic species, for the “Mean trapping length” response variable. Estimated effects ± standard errors are given, with corresponding t and p values. The factor “Family”, an amalgamation of family and super family was used as random factor, and effects given are standard deviations across groups.

|  | All species | | | Saproxylic species | | |
| --- | --- | --- | --- | --- | --- | --- |
|  | Effect ± sd | t | p | Effect ± sd | t | p |
| *Fixed effects* |  |  |  |  |  |  |
| Intercept | -104.3 ± 57.7 | -1.81 | 0.071 | -82.4 ± 65.3 | -1.26 | 0.208 |
| Proportional abundance (log transformed) | 3.2 ± 0.4 | 7.74 | <0.001 | 2.6 ± 0.5 | 5.46 | <0.001 |
| Size (log transformed) | -4.5 ± 0.8 | -5.35 | <0.001 | -4.4 ± 1.0 | -4.35 | <0.001 |
| Latitude | 3.3 ± 1.0 | 3.43 | <0.001 | 2.9 ± 1.1 | 2.66 | 0.008 |
| Latitudinal range | 4.1 ± 2.5 | 1.66 | 0.097 | 6.6 ± 2.9 | 2.31 | 0.021 |
| df | 556 |  |  | 392 |  |  |
| *Random effects* |  |  |  |  |  |  |
| Intercept | 1.1 |  |  | 2.38 |  |  |
| Family | 0.006 |  |  | 0.03 |  |  |
| Rsq-adj | 0.18 |  |  | 0.18 |  |  |

Table S3.2: Parameter estimates of the two linear mixed models performed using the full dataset and the subset of Saproxylic species, for the “weighted month standard deviation” response variable. Estimated effects ± standard errors are given, with corresponding t and p values. The factor “Family”, an amalgamation of family and super family was used as random factor, and effects given are standard deviations across groups.

|  | All species | | | Saproxylic species | | |
| --- | --- | --- | --- | --- | --- | --- |
|  | Effect ± sd | t | p | Effect ± sd | t | p |
| *Fixed effects* |  |  |  |  |  |  |
| Intercept | 0.6 ± 0.6 | 0.88 | 0.379 | 0.7 ± 0.6 | 1.11 | 0.269 |
| Proportional abundance (log transformed) | 0.01 ± 0.0 | 3.59 | <0.001 | 0.01 ± 0.0 | 2.58 | 0.010 |
| Size (log transformed) | -0.04 ± 0.0 | -4.60 | <0.001 | -0.04 ± 0.0 | -4.10 | <0.001 |
| Latitude | 0.00 ± 0.0 | 0.18 | 0.856 | -0.00 ± 0.0 | -0.10 | 0.919 |
| Latitudinal range | 0.1 ± 0.0 | 2.94 | 0.003 | 0.1 ± 0.0 | 2.95 | 0.003 |
| df | 556 |  |  | 392 |  |  |
| *Random effects* |  |  |  |  |  |  |
| Intercept | 0.02 |  |  | 0.03 |  |  |
| Family | 0.02 |  |  | 0.05 |  |  |
| Rsq-adj | 0.1 |  |  | 0.12 |  |  |

Table S3.3: Parameter estimates of the two linear mixed models performed using the full dataset and the subset of Saproxylic species, for the “Seasonality index” response variable. Estimated effects ± standard errors are given, with corresponding t and p values. The factor “Family”, an amalgamation of family and super family was used as random factor, and effects given are standard deviations across groups. Note that the sign of the effects are reversed for this variable: e.g., there is a negative effects of abundance on seasonality, because a low index value corresponds to a long flight period.

|  | All species | | | Saproxylic species | | |
| --- | --- | --- | --- | --- | --- | --- |
|  | Effect ± sd | t | p | Effect ± sd | t | p |
| *Fixed effects* |  |  |  |  |  |  |
| Intercept | -0.56 ± 0.6 | -0.95 | 0.341 | -0.26 ± 0.6 | -0.42 | 0.672 |
| Proportional abundance (log transformed) | -0.02 ± 0.0 | -3.83 | <0.001 | -0.01 ± 0.0 | -2.78 | 0.006 |
| Size (log transformed) | 0.04 ± 0.0 | 4.06 | <0.001 | 0.05 ± 0.0 | 5.48 | <0.001 |
| Latitude | 0.02 ± 0.0 | 1.98 | 0.049 | 0.01 ± 0.0 | 1.36 | 0.173 |
| Latitudinal range | -0.05 ± 0.0 | -2.17 | 0.030 | -0.05 ± 0.0 | -1.96 | 0.051 |
| df | 556 |  |  | 392 |  |  |
| *Random effects* |  |  |  |  |  |  |
| Intercept | 0.03 |  |  | 0.02 |  |  |
| Family | 0.02 |  |  | 0.03 |  |  |
| Rsq-adj | 0.1 |  |  | 0.13 |  |  |

**Appendix S4: Parameter estimates of single site analysis**

Table S4 Parameter estimates of the linear mixed models performed using data for all years from a single site, for the flight activity period response variable. Estimated effects ± standard errors are given, with corresponding t and p values.

|  | Effect ± sd | df | t | p |
| --- | --- | --- | --- | --- |
| *Fixed effects* |  |  |  |  |
| Intercept | 20.5 ± 18.1 | 382 | 1.13 | 0.258 |
| GDD | 0.04 ± 0.04 | 5 | 1.02 | 0.310 |
| Size (log transformed) | 10.1 ± 5.5 | 382 | 1.83 | 0.068 |
| Presence | 4.2 ± 0.4 | 382 | 10.20 | <0.001 |
| GDD x size | -0.03 ± 0.01 | 382 | -2.26 | 0.024 |
| *Random effects* |  |  |  |  |
| Intercept (Year) | 5.99 |  |  |  |
| Intercept (Family) | 2.32 |  |  |  |
| Intercept (Species) | 10.7 |  |  |  |
| Residual | 5.08 |  |  |  |
| Rsq-adj | 0.09 |  |  |  |
